# Supplementary material for: School-based physical activity interventions among children and adolescents in the Middle East and Arabic speaking countries: A systematic review
Source: PLoS One. 2023 Jul 3;18(7):e0288135. doi: 10.1371/journal.pone.0288135 (PMC10317243; doi:10.1371/journal.pone.0288135)
Supplement: S1 File — (DOCX) [file pone.0288135.s002.docx]

**Appendix 1. Search strategy.**

(“School-based intervention*” OR School* OR “School-based program*” OR “School-based”) AND (“Exercise*” OR “Physical activit*” OR “physical exercise” OR “Aerobic Exercise”) AND (“child*” OR “Adolescent*” OR Teen* OR “Young people” OR Youth)

AND

(Yemen OR United Arab Emirates OR Syria OR Saudi Arabia OR Qatar OR Oman OR Lebanon OR Kuwait OR Jordan OR Bahrain OR Palestinian OR Tunisia OR Egypt OR Libya OR Morocco OR Comoros OR Djibouti OR Mauritania OR Algeria OR Sudan OR Somalia OR Iraq OR Israel OR Iran OR Turkey OR Cyprus)

**Appendix 2. Risk Assessment CASP Tool for RCTs.**

| **Items** | **Taymoori & Lubans (2008)** | **El Ansari et al. (2010)** | **Saffari et al. (2013)** | **Habib-Mourad et al. (2014)** | **Darabi et al. (2017)** | **Rostami-Moez et al. (2017)** | **Simbar et al. (2017)** | **Stanley et al. (2017)** | **Habib-Mourad et al. (2020)** | **Habib-Mourad et al. (2020a)** | **Allafi 2020** | | Bahathig & Saad (2022) | **Kutbi et al., 2019** |
| --- | --- | --- | --- | --- | --- | --- | --- | --- | --- | --- | --- | --- | --- | --- |
| Item 1 | Yes | Yes | Yes | Yes | Yes | Yes | Yes | Yes | Yes | Yes | Yes | | Yes | Yes |
| Item 2 | Yes | Yes | Unclear | Yes | Yes | Yes | Yes | Yes | Yes | Yes | Yes | | Yes | Yes |
| Item 3 | Yes | Yes | Unclear | Yes | Yes | Yes | Yes | Yes | Yes | Yes | Yes | | Yes | Yes |
| Item 4 | Unclear | Unclear | Unclear | Unclear | Unclear | Unclear | Unclear | Unclear | Unclear | Unclear | Unclear | | Unclear | Unclear |
| Item 5 | Yes | Yes | Yes | Yes | Yes | Yes | Yes | Yes | Yes | Yes | Yes | | Yes | Yes |
| Item 6 | Yes | Yes | Yes | Yes | Yes | Yes | Yes | Yes | Yes | Yes | Yes | | Yes | Yes |
| Item 7 | Primary outcome was clearly specified – *p*-value (< .05 | Primary outcome was clearly specified – *p*-value (< .05 | Primary outcome was clearly specified – *p*-value (< .001 | Primary outcome was clearly specified – *p*-value (< .001 | Primary outcome was clearly specified – *p*-value (< .001 | Primary outcome was clearly specified – *p*-value (< .01 | Primary outcome was clearly specified – *p*-value (< .05 | Primary outcome was clearly specified – *p*-value (< .05 | Primary outcome was clearly specified – *p*-value (< .01 | Primary outcome was clearly specified – *p*-value (< .05 | Primary outcome was clearly specified – *p*-value (< 0.05) | Primary outcome was clearly specified  (p > 0.05) | | Primary outcome was clearly specified – *p*-value (< .05) |
| Item 8 | Yes – 95% Confidence limit applied | Yes – 95% Confidence limit applied / correlation coefficient was .97% | Yes – 95% Confidence limit applied | Yes – 95% Confidence limit applied | Yes – 95% Confidence limit applied | Yes – 95% Confidence limit applied / correlation coefficient was .86% | Yes – 95% Confidence limit applied | Yes – 95% Confidence limit applied | Yes – 95% Confidence limit applied | Yes – 95% Confidence limit applied | Yes – 95% Confidence limit applied | Yes – 95% Confidence limit applied | | Yes – 95% Confidence limit applied |
| Item 9 | Yes | Yes | Yes | Yes | Yes | Yes | Yes | Yes | Yes | Yes | Yes | Yes | | Yes |
| Item 10 | Yes | Yes | Unclear – the | Yes | Yes | Yes | Yes | Unclear | Yes | Yes | Yes | Yes | | Yes |
| Item 11 | Yes | Yes | Yes | Yes | Yes | Yes | Yes | Yes | Yes | Yes | Yes | Yes | | Yes |

*Note:*

***Item 1****: Did the trial address a clearly focused issue?* ***Item 2****: Was the assignment of patients to treatments randomised?* ***Item 3****: Were all of the patients who entered the trial properly accounted for at its conclusion?* ***Item 4****: Were patients, health workers and study personnel ‘blind’ to treatment?* ***Item 5****: Were the groups similar at the start of the trial?* ***Item 6****: Aside from the experimental intervention, were the groups treated equally?* ***Item 7****: How large was the treatment effect?* ***Item 8****: How precise was the estimate of the treatment effect?* ***Item 9****: Can the results be applied to the local population, or in your context?* ***Item 10****: Were all clinically important outcomes considered?* ***Item 11****: Are the benefits worth the harms and costs?*

**Appendix 3. JBI Quasi-Experimental Studies Tool**

| **Item / Study** | **Maatoug et al. (2013)** | **Maatoug et al. (2015)** | **Ghamman et al. (2017)** | **Elfaki et al., 2020** |
| --- | --- | --- | --- | --- |
| 1) Is it clear in the study what is the ‘cause’ and what is the ‘effect’ (i.e. there is no confusion about which variable comes first)? | Yes | Yes | Yes | Yes |
| 2) Were the participants included in any comparisons similar? | Yes | Yes | Yes | Yes |
| 3) Were the participants included in any comparisons receiving similar treatment/care, other than the exposure or intervention of interest? | Yes | Yes | Yes | Yes |
| 4) Was there a control group? | Yes | Yes | Yes | Yes |
| 5) Were there multiple measurements of the outcome both pre and post the intervention/exposure? | Yes | Unclear | Yes | Yes |
| 6) Was follow up complete and if not, were differences between groups in terms of their follow up adequately described and analyzed? | Yes | Unclear | Yes | Unclear |
| 7) Were the outcomes of participants included in any comparisons measured in the same way? | Unclear | Unclear | Unclear | Unclear |
| 8) Were outcomes measured in a reliable way? | Yes | Yes | Yes | Yes |
| 9) Was appropriate statistical analysis used? | Yes | Yes | Yes | Yes |

**Appendix 4. Studies were excluded during full-text screening with reasons.**

**Not school-based intervention (14)**

Abeer, A. S., Saniya, S., Mona, A. T., Buthiania, A. S., & Mariam, A. A. (2015). Interventional program for teenagers' obesity.

Aljuhani, O. and Sandercock, G., 2019. Contribution of physical education to the daily physical activity of schoolchildren in Saudi Arabia. *International journal of environmental research and public health*, *16*(13), p.2397.

Alharbi, M., 2019. Influence of individual and family factors on physical activity among Saudi girls: a cross-sectional study. *Annals of Saudi medicine*, *39*(1), pp.13-21.

Boodai, S. A., McColl, J. H., & Reilly, J. J. (2014). National Adolescent Treatment Trial for Obesity in Kuwait (NATTO): project design and results of a randomised controlled trial of a good practice approach to treatment of adolescent obesity in Kuwait. *Trials*, *15*(1), 1-6.

Al-Thani, M., Al-Thani, A., Alyafei, S., Al-Kuwari, M.G., Al-Chetachi, W., Khalifa, S.E., Ibrahim, I., Sayegh, S., Vinodson, B. and Akram, H., 2018. Prevalence of physical activity and sedentary-related behaviors among adolescents: data from the Qatar National School Survey. *Public Health*, *160*, pp.150-155.

Zimmo, L., Farooq, A., Almudahka, F., Ibrahim, I. and Al-Kuwari, M.G., 2017. School-time physical activity among Arab elementary school children in Qatar. *BMC pediatrics*, *17*(1), pp.1-7.

Pengpid, S. and Peltzer, K., 2020. Trends in the prevalence of twenty health indicators among adolescents in United Arab Emirates: cross-sectional national school surveys from 2005, 2010 and 2016. *BMC pediatrics*, *20*(1), pp.1-11.

Al-Domi, H.A., Faqih, A., Jaradat, Z., Anfal, A.D., Jaradat, S. and Amarneh, B., 2019. Physical activity, sedentary behaviors and dietary patterns as risk factors of obesity among Jordanian schoolchildren. *Diabetes & Metabolic Syndrome: Clinical Research & Reviews*, *13*(1), pp.189-194.

Al Husaini, A. (2017). Physical Activity and Sedentary Behavior Relative to Body Mass Index among School Children in Saudi Arabia. *Pediatr Ther*, *7*(312), 2161-0665.

Althumiri, N. A., BinDhim, N. F., & Alqahtani, S. A. (2020). Prevalence of physical inactivity and sedentary behaviors and associations with obesity among Saudi Adults. *J Health Econ Outcomes Res*.

Al Eid, A. J., Alahmed, Z. A., Al-Omary, S. A., & Alharbi, S. M. (2017). RASHAKA Program: A collaborative initiative between Ministry of Health and Ministry of Education to control childhood obesity in Saudi Arabia. *Saudi Journal of Obesity*, *5*(1), 22.

Alnasser, A., Kyle, J., Aloumi, N., Al-Khalifa, A., & Marais, D. (2019). The Twazon Arabic Weight Loss App: App-Based Intervention for Saudi Women With Obesity. *JMIR mHealth and uHealth*, *7*(5), e10923.

Alturki, H. A., Brookes, D. S., & Davies, P. S. (2018). Obesity prevention interventions in Saudi Arabian children–building the evidence base: An in-depth analysis of sociodemographic characteristics and dietary habits of obese and normal weight schoolchildren. *Glob Epidemic Obes*, *6*.

Al-Hazzaa, H. M. (2007). Pedometer-determined physical activity among obese and non-obese 8-to 12-year-old Saudi schoolboys. *Journal of physiological anthropology*, *26*(4), 459-465.‏‏‏‏‏‏

**Having an inappropriate target population (2)**

Nemet, D., Geva, D., Pantanowitz, M., Igbaria, N., Meckel, Y., & Eliakim, A. (2013). Long term effects of a health promotion intervention in low socioeconomic Arab-Israeli kindergartens. *BMC pediatrics*, *13*(1), 1-7.

Kaufman-Shriqui, V., Fraser, D., Friger, M., Geva, D., Bilenko, N., Vardi, H., ... & Shahar, D. R. (2016). Effect of a school-based intervention on nutritional knowledge and habits of low-socioeconomic school children in Israel: a cluster-randomized controlled trial. *Nutrients*, *8*(4), 234.‏

‏‏

**Studies focusing only on obese and overweight students (5)**

Haghani, S., Shahnazi, H., & Hassanzadeh, A. (2017). Effects of tailored health education program on overweight elementary school students’ obesity-related lifestyle: A school-based interventional study. *Oman medical journal*, *32*(2), 140.

Hassan, N. E. M., Zaki, S. T., El-masry, S., Mohsen, M. A., & Elashmawy, E. (2011). Impact of Balanced Caloric Diet and Physical activity on Body Compo-sition and Fat Distribution of Obese Egyptian Adolescent Girls. *Maced J Med Sci*, *4*(1), 17-24.

Amini, M., Djazayery, A., Majdzadeh, R., Taghdisi, M. H., Sadrzadeh-Yeganeh, H., Abdollahi, Z., ... & Nourmohammadi, M. (2016). A school-based intervention to reduce excess weight in overweight and obese primary school students. *Biological research for nursing*, *18*(5), 531-540.

Bagherniya, M., Darani, F. M., Sharma, M., Maracy, M. R., Birgani, R. A., Ranjbar, G., ... & Keshavarz, S. A. (2018). Assessment of the efficacy of physical activity level and lifestyle behavior interventions applying social cognitive theory for overweight and obese girl adolescents. *Journal of research in health sciences*, *18*(2), 409.

Aljassim, H. and Jradi, H., 2021. Childhood overweight and obesity among the Saudi population: a case-control study among school children. *Journal of Health, Population and Nutrition*, *40*(1), pp.1-9.

**Inappropriate study type (7) = 15**

Habib-Mourad, C., & Ghandour, L. A. (2015). Time to act: lessons learnt from the first pilot school-based intervention study from Lebanon to prevent and reduce childhood obesity. *Frontiers in public health*, *3*, 56.

Al-Haifi, A. R., Al-Fayez, M. A., Al-Nashi, B., Al-Athari, B. I., Bawadi, H., & Musaiger, A. O. (2012). Right Diet: a television series to combat obesity among adolescents in Kuwait. *Diabetes, metabolic syndrome and obesity: targets and therapy*, *5*, 205.

Musaiger, A. O., Al Hazzaa, H. M., Al-Qahtani, A., Elati, J., Ramadan, J., AboulElla, N. A., ... & Kilani, H. A. (2011). Strategy to combat obesity and to promote physical activity in Arab countries. *Diabetes, metabolic syndrome and obesity: targets and therapy*, *4*, 89.

Bajamal, E., Robbins, L.B., Ling, J., Smith, B., Pfeiffer, K.A. and Sharma, D., 2017. Physical activity among female adolescents in Jeddah, Saudi Arabia: A health promotion model-based path analysis. *Nursing research*, *66*(6), pp.473-482.

Baniissa, W., Radwan, H., Rossiter, R., Fakhry, R., Al-Yateem, N., Al-Shujairi, A., ... & Awad, M. A. (2020). Prevalence and determinants of overweight/obesity among school-aged adolescents in the United Arab Emirates: a cross-sectional study of private and public schools. *BMJ open*, *10*(12), e038667.

Aperman-Itzhak, T., Yom-Tov, A., Vered, Z., Waysberg, R., Livne, I., & Eilat-Adar, S. (2018). School-Based intervention to promote a healthy lifestyle and obesity prevention among fifth-and sixth-grade children. *American Journal of Health Education*, *49*(5), 289-295.

Benajiba, N., Mahrous, L., Janah, K., Alqabbani, S. F., Chavarria, E. A., & Aboul‐Enein, B. H. (2020). Physical activity interventions promoted in the Arabic‐speaking region: a review of the current literature. *Obesity Reviews*, *21*(8), e13032.

‏

‏
