# Supplementary material for: School-based physical activity interventions among children and adolescents in the Middle East and Arabic speaking countries: A systematic review
Source: PLoS One. 2023 Jul 3;18(7):e0288135. doi: 10.1371/journal.pone.0288135 (PMC10317243; doi:10.1371/journal.pone.0288135)
Supplement: Attachment — Submitted filename: Review paper Plos One (PONE-D-23-05274) 20.3.23.docx [file pone.0288135.s003.docx]

**Review of Manuscript for PLOS ONE**

Manuscript number: PONE-D-23-05274

Thank you for the opportunity to review the paper entitled*: ‘School-based physical activity interventions among children and adolescents in the Middle East and Arabic speaking countries: a systematic review.*

The manuscript addresses an important gap in the literature in relation to children’s physical activity in Arabic and Middle Eastern countries. The paper is reasonably well written but there are several typographical and grammatical errors throughout. It is not clear if all the PRISMA guidelines were adhered to, please see my comments in the methods section of this review. I have listed several suggestions below. I hope the authors find the comments helpful and wish them all the best with their future research.

**Abstract**

i) Page 3 – Conclusion- check grammar in section “multi-component includes education”

**Introductory section**

i) Please add references to all sentences which do not have a reference in the introduction section of the manuscript.
ii) I wonder, given that this article solely focuses on children, it might be better to acknowledge in one sentence the health impact of low levels of PA on the broader population (e.g., adults) but then focus more specifically on the health impact of low levels of PA in children, as there is quite a lot of literature in this area now.
iii) Start of second paragraph suggest using ‘increase’ rather than ‘raise’
iv) Some of the references used are quite old and should be updated – suggest reviewing all references that are older than 10 years.
v) Suggest indicating what the PA levels are in Arabic countries to provide more insight in the sentence: More specifically, the average level of PA is low in the Arabic speaking countries, compared with other countries [7].
vi) Last sentence of paragraph 2 - Suggest that exercise and physical activity are not used interchangeably, as people can still be physically active without exercising. Suggest checking the manuscript throughout and changing where necessary.
vii) Suggest that physical inactivity is not used interchangeably with sedentary behaviour. As being physically inactive means that someone is not doing enough physical activity and being sedentary means that they are sitting or lying down for long periods.
viii) Last sentence on page 5 - suggest changing the word ‘find’ to: understand

**Methods**

i) Did you use the PRISMA framework for systematic reviews? If you used the PRISMA guidelines, suggest that this be added to the methods and abstract.
ii) Page 7 - Add ‘the’ to the sentence commencing: Search parameters were limited to ‘the’ English language.
iii) In the section entitled: Search Strategy – I suggest combining individual sentences into one paragraph. I am just wondering why the Arabic studies data bases were not included in the search.

*Selection of studies*

iv) The study selection does not seem to have followed the PRISMA guidelines where 2 to 3 reviewers independently select relevant studies.
v) Was a referencing packaged used (e.g., Endnote)? Please describe how the references were managed.
vi) MENA should be written in full when first cited in the paper with MENA in brackets.

*Study characteristics*

vii) Page 10 second paragraph second sentence. Note: 8-year-olds are not generally considered adolescents – those 13 years and above are adolescents. Suggest revising this section

*Risk of Bias*

viii) Page 16 first sentence suggest adding ‘an’ to the following part of the sentence had ‘an’ unclear risk of bias.
ix) Page 16 first paragraph second last sentence: Suggest rewording this sentence to state that selective bias was low in ten trials and unclear in two trials …..
x) In relation to the above sentence – it seems that one trial is missing??
xi) Page 16 second paragraph. This paragraph includes one very long sentence – suggest revising to at least two sentences.
xii) Page 16 - suggest providing more details of the relevant studies in relation to the following sentence: Nonetheless, it is important to note that the outcomes seemed reliable judging by the number of raters and the intra-rater and inter-raters reliabilities where reported.

*Data synthesis*

xiii) Page 17 second sentence check grammar for ‘These includes’
xiv) Page 17 – need a full stop in this sentence: PA levels and social support differed significantly between males and females According to the research, during the intervention…
xv) Also suggest removing the word ‘an’ from this sentence: This study [27] reported an changes in knowledge..
xvi) Page 18 check grammar in this sentence: Engaging in PA, on the other hand, was used in only two study

And

Page 18 The two study focusing

xvii) I am not 100% sure of the journal formatting but suggest that the authors names should be referred to in sections on page 19 where it just uses numbers e.g., alone (22) reported….
xviii) Page 20 add ‘the’ to this sentence: In [17], both PA only

**Discussion**

i) Page 21 second paragraph of the discussion. It is unclear which studies are being referred to in this paragraph. Were the four remaining studies a part of the first study? Suggest revising and adding references to this paragraph.
ii) Also suggest adding more detail in relation to the following sentence from the above paragraph: related to factors such as the content and details of the school-based intervention programme.
iii) Page 22 suggest adding ‘the’ to the sentence: to consider potential for PA other times.
iv) Page 22 third paragraph suggest adding ‘that’ to the following sentence: schools have not been implemented to any extent
v) Page 23 first paragraph suggest adding ‘that’ to this sentence: strong evidence school-based PA interventions
vi) Page 23 paragraph 1 – Suggest revising the last sentence for grammar.
vii) Page 23 paragraph 2 -suggest revising this part of the sentence: ‘met inclusion criteria’ to read: that met the inclusion criteria
viii) Page 23 paragraph 2 Suggest expanding a little on the following sentence so that the reader understands the focus of these reviews in comparison to the current review in the section: These results align with those of Nash et al. and Benajiba et al., which recently focused on interventions to promote physical activity in the region [43,44].

**Implications for research**

i) Page 25 first paragraph: suggest revising this sentence: ‘have potential in increasing PA levels’ to read have the potential to increase PA levels
ii) Page 25 paragraph two suggest keeping the PA focus and stating: favourable benefits of PA on health in general

and

iii) Suggest removing capitalization on the word: Standardised.

**Conclusion**

Suggest revising the conclusion for grammatical errors.

i) Page 26: conclusion – check grammar in this section: ‘who increase participate in physical activity levels’.

And this section:

ii) To date, limited range of types few evaluations focused solely on assess PA specific interventions and most of the interventions were multi-component (includes education components on lifestyle, and diet, education).

And

iii) An ‘r’ is missing from this section: ‘have been effective in other egions’
