# Supplementary material for: School-based physical activity interventions among children and adolescents in the Middle East and Arabic speaking countries: A systematic review
Source: PLoS One. 2023 Jul 3;18(7):e0288135. doi: 10.1371/journal.pone.0288135 (PMC10317243; doi:10.1371/journal.pone.0288135)
Supplement: Attachment — Submitted filename: Response to Reviewers.docx [file pone.0288135.s004.docx]

**A response to the reviewer’s comments point by point and how we addressed them:**

**Reviewer #1 Comments:**

Dear authors, the research topic is very current. The manuscript is methodologically very well written, in accordance with the guidelines for writing review research. The manuscript has some details that need fixing:

1. The first sentence in the Introduction requires a citation

Citation added.

2. in the section Selection of studies, it is unnecessary to state the author's initials, this part should be written without a specific indication of the author

Changed to:

All records are exported to Mendeley reference manager software. Duplicate records and all studies whose title showed did not meet the inclusion were excluded. Screening of studies was conducted by two reviewers independently, titles and abstracts of records retrieved from searches were screened for inclusion by two of them, and any disagreement was resolved by discussion with a third reviewer.

3. In Figure 1, the number 1349 needs to be technically corrected

Corrected.

4. #the combined number of school-aged children who took part in the 17 studies was 15,551 with a mean sample size of 1,113" - I am not clear about this average? That is not correct information! And it is not necessary because each study has its own number of subjects.

This sentence was deleted as suggested.

**Reviewer #2 Comments:**

The manuscript addresses an important gap in the literature in relation to children’s physical activity in Arabic and Middle Eastern countries. The paper is reasonably well written but there are several typographical and grammatical errors throughout. It is not clear if all the PRISMA guidelines were adhered to, please see my comments in the methods section of this review. I have listed several suggestions below. I hope the authors find the comments helpful and wish them all the best with their future research.

**Abstract**
i) Page 3 – Conclusion- check grammar in section “multi-component includes education”

Changed to (were multi-component including education).

**Introductory section**

i) Please add references to all sentences which do not have a reference in the introduction section of the manuscript.

Revised and added successfully.

ii) I wonder, given that this article solely focuses on children, it might be better to acknowledge in one sentence the health impact of low levels of PA on the broader population (e.g., adults) but then focus more specifically on the health impact of low levels of PA in children, as there is quite a lot of literature in this area now.

This sentence added

In developed countries, current levels of PA participation are generally lower than recommendations for gaining health benefits for both adults and children [7,8]. With specific regard to children and adolescents…

iii) Start of second paragraph suggest using ‘increase’ rather than ‘raise’

Changed to increase.

iv) Some of the references used are quite old and should be updated – suggest reviewing all references that are older than 10 years.

All references updated as suggested.

v) Suggest indicating what the PA levels are in Arabic countries to provide more insight in the sentence: More specifically, the average level of PA is low in the Arabic speaking countries, compared with other countries [7].

More specifically, Chaabane et. al. [10] reported that Arabic countries has the highest concentration of non-communicable diseases in the world and have the second-highest prevalence of diabetes worldwide (10.9%) [9] and they are recording a speedy increase in the level of obesity [10.11]. This low level of PA is one of the factors that has contributed to a pandemic of chronic diseases in the region, such as heart disease, obesity and diabetes [12].

vi) Last sentence of paragraph 2 - Suggest that exercise and physical activity are not used interchangeably, as people can still be physically active without exercising. Suggest checking the manuscript throughout and changing where necessary.

Changed all to physical activity.

vii) Suggest that physical inactivity is not used interchangeably with sedentary behaviour. As being physically inactive means that someone is not doing enough physical activity and being sedentary means that they are sitting or lying down for long periods.

Changed to:

Children and adolescents who are not doing enough physical activity are more vulnerable to energy imbalance…

viii) Last sentence on page 5 - suggest changing the word ‘find’ to: understand

Changed to understand.

**Methods**

i) Did you use the PRISMA framework for systematic reviews? If you used the PRISMA guidelines, suggest that this be added to the methods and abstract.

Added successfully to abstract and method section.

ii) Page 7 - Add ‘the’ to the sentence commencing: Search parameters were limited to ‘the’ English language.

Added successfully.

iii) In the section entitled: Search Strategy – I suggest combining individual sentences into one paragraph. I am just wondering why the Arabic studies data bases were not included in the search.

Combined as suggested.

There is no study in Arabic databases has been found and we indicated that (The search parameters were limited to English and Arabic language publications published between January 2000 and January 2023.)

**Selection of studies**

iv) The study selection does not seem to have followed the PRISMA guidelines where 2 to 3 reviewers independently select relevant studies.

Changed to:

Changed to:

All records are exported to Mendeley reference manager software. Duplicate records and all studies whose title showed did not meet the inclusion were excluded. Screening of studies was conducted by two reviewers independently, titles and abstracts of records retrieved from searches were screened for inclusion by two of them, and any disagreement was resolved by discussion with a third reviewer.

v) Was a referencing packaged used (e.g., Endnote)? Please describe how the references were managed.

Please see the comment above.

vi) MENA should be written in full when first cited in the paper with MENA in brackets.

Added successfully. The Middle East and North Africa (MENA).

**Study characteristics**

vii) Page 10 second paragraph second sentence. Note: 8-year-olds are not generally considered adolescents – those 13 years and above are adolescents. Suggest revising this section

Changed to (All the studies focused on school-aged children as their target population with a range from 8 years [34] to 16 years).

**Risk of Bias**

viii) Page 16 first sentence suggest adding ‘an’ to the following part of the sentence had ‘an’ unclear risk of bias.

Added successfully

ix) Page 16 first paragraph second last sentence: Suggest rewording this sentence to state that selective bias was low in ten trials and unclear in two trials …..

Changed to:

Furthermore, selective bias was low in eleven trials and unclear in only two trials [28,33] because they did not report all the outcomes that they said they measured.

x) In relation to the above sentence – it seems that one trial is missing??

Yes, added.

xi) Page 16 second paragraph. This paragraph includes one very long sentence – suggest revising to at least two sentences.

Changed to two sentences.

xii) Page 16 - suggest providing more details of the relevant studies in relation to the following sentence: Nonetheless, it is important to note that the outcomes seemed reliable judging by the number of raters and the intra-rater and inter-raters reliabilities where reported.

Relevant studies cited.

**Data synthesis**

xiii) Page 17 second sentence check grammar for ‘These includes’

Corrected to be (These include).

xiv) Page 17 – need a full stop in this sentence: PA levels and social support differed significantly between males and females According to the research, during the intervention…

a full stop was added.

xv) Also suggest removing the word ‘an’ from this sentence: This study [27] reported an changes in knowledge..

removed ‘an’

xvi) Page 18 check grammar in this sentence: Engaging in PA, on the other hand, was used in only two study
And
Page 18 The two study focusing

changed to (On the other hand, engaging in PA was used in only two studies [27,37].

xvii) I am not 100% sure of the journal formatting but suggest that the authors names should be referred to in sections on page 19 where it just uses numbers e.g., alone (22) reported…

Authors names added

xviii) Page 20 add ‘the’ to this sentence: In [17], both PA only

added

**Discussion**

i) Page 21 second paragraph of the discussion. It is unclear which studies are being referred to in this paragraph.

Were the four remaining studies a part of the first study? Suggest revising and adding references to this paragraph.

For the remaining four studies [28,32,33,38],

iii) Page 22 suggest adding ‘the’ to the sentence: to consider potential for PA other times.

Changed to (to consider the potential for PA at other times).

iv) Page 22 third paragraph suggest adding ‘that’ to the following sentence: schools have not been implemented to any extent

added

v) Page 23 first paragraph suggest adding ‘that’ to this sentence: strong evidence school-based PA interventions

added

vi) Page 23 paragraph 1 – Suggest revising the last sentence for grammar.

However, it should be noted that due to unique climatic, social, and cultural factors in Arabic-speaking countries, it is unlikely that interventions developed elsewhere will be applied directly or without modification to those countries.

vii) Page 23 paragraph 2 -suggest revising this part of the sentence: ‘met inclusion criteria’ to read: that met the inclusion criteria

changed as suggested.

viii) Page 23 paragraph 2 Suggest expanding a little on the following sentence so that the reader understands the focus of these reviews in comparison to the current review in the section: These results align with those of Nash et al. and Benajiba et al., which recently focused on interventions to promote physical activity in the region [43,44].

Added

**Implications for research**

i) Page 25 first paragraph: suggest revising this sentence: ‘have potential in increasing PA levels’ to read have the potential to increase PA levels

Changed successfully.

ii) Page 25 paragraph two suggest keeping the PA focus and stating: favourable benefits of PA on health in general.

Changed successfully.

iii) Suggest removing capitalization on the word: Standardised.

Removed as suggested.

**Conclusion**
Suggest revising the conclusion for grammatical errors.

Revised.

i) Page 26: conclusion – check grammar in this section: ‘who increase participate in physical activity levels’.
And this section:

Corrected. participation

ii) To date, limited range of types few evaluations focused solely on assess PA specific interventions and most of the interventions were multi-component (includes education components on lifestyle, and diet, education).
And

a limited… assessing … (including…

iii) An ‘r’ is missing from this section: ‘have been effective in other egions’.

Corrected to (regions)

**Thank you so much for your valuable suggestions.**
