# Supplementary material for: School-based physical activity interventions among children and adolescents in the Middle East and Arabic speaking countries: A systematic review
Source: PLoS One. 2023 Jul 3;18(7):e0288135. doi: 10.1371/journal.pone.0288135 (PMC10317243; doi:10.1371/journal.pone.0288135)
Supplement: Attachment — Submitted filename: Response to Reviewers.docx [file pone.0288135.s005.docx]

**A response to the reviewer’s comments point by point and how we addressed them:**

**Reviewer #1 Comments:**

Reviewer #1: All comments from the reviewers have been accepted and the manuscript has now been reconciled with the comments submitted.

**Reviewer #3:** This was an interesting review that explores physical activity interventions across Arabic-speaking countries. The manuscript is certainly unique in its own right, however, there are some major concerns worth raising.
My primary concern with this review involves the Methods section.
Firstly, the use of additional electronic academic databases would likely have added to the study in both complexity and sample size. If the scope of this study were expanded to use additional databases, more sources might have been identified and explored. For example, the following electronic databases could have been used for a more thorough and inclusive search;
“ArticleFirst; Biomed Central; BioOne; BIOSIS; EBSCOHost; JSTOR; ProQuest; SAGE Reference Online; ScienceDirect; SpringerLink; Taylor & Francis; and Wiley Online.” These databases would have likely added to the overall literature search in their academic rigor, aim, and biomedical scope. While PubMed/MEDLINE, Web of Science, Scopus and CINAHL is an excellent database, the use of more databases would have added to the study sample size. Also, I recommend adding French in the language search as well. Not using French would potentially neglect countries like Morocco, Tunisia, Algeria etc. as part of the search parameters.

We appreciate the concern that the limitations of our database search strategy and restriction to English language articles may have led to our searches not being exhaustive but were constrained by resource considerations as this review was undertaken by the first author within a PhD programme. We have mitigated these limitations in several ways including forward and backwards (citation and reference) searching and checking the reference lists of other related systematic reviews. These strategies provide reassurance that it is unlikely that there is a substantive number of additional studies which would meet our inclusion criteria and change our overall conclusions about the nature of current evidence in this field. We have added a more detailed explanation in the Discussion under “Limitations” on the specific limitations and associated mitigating strategies.

“Despite the limitations of our initial database searches which were constrained by available resources, we have mitigated these limitations in several ways including forward and backwards (citation and reference) searching and checking the reference lists of other related systematic reviews. These strategies provide reassurance that it is unlikely that there is a substantive number of additional studies which would meet our inclusion criteria and change our overall conclusions about the nature of current evidence in this field.”

Secondly, the Arab world (or the Middle east) should be clearly and expressly defined. I strongly suggest the authors review this article thoroughly and clearly define what ‘Arabic-speaking countries’ are. What defines the ‘Middle East’? Israel (Arabic is a recognized language in Israel), Iran, and Turkey may be argued as part of the Middle East…were those counted as part of the search parameters. I recommend using the Arab League member states as the definition. This should be written clearly in the methods section. I suggest citing the article too to improve the merit of this review.
<https://www.ncbi.nlm.nih.gov/pmc/articles/PMC3988371/>

Thank you for this suggestion; we recognize the need for clarification of both the inclusion criteria and the rationale for the choice of countries included as this is important for the interpretation of our findings and their generalizability to other countries in the region.

We have therefore added additional clarification on pages 5 and 7 of the manuscript

Methods:

“We included Arabic-speaking countries located in the Arabian Peninsula, northern parts of Africa, and the East Mediterranean [13]. It contains 22 countries ((i.e., Yemen, United Arab Emirates, Syria, Saudi Arabia, Qatar, Oman, Lebanon, Kuwait, Jordan, Bahrain, Palestinian, Tunisia, Egypt, Libya, Morocco, Comoros, Djibouti, Mauritania, Algeria, Sudan, Somalia, and Iraq). These countries are defined as the 22 member countries of the League of Arab States [14]; along with countries in the Middle East that are not counted in the Arabic-speaking countries (i.e., Iran, Israel, Turkey, and Cyprus).”

Discussion (under Strengths):

“We included both Arabic speaking countries and additional countries in the same region (Iran, Israel, Turkey, and Cyprus) to maximise the number of potentially relevant studies included in the review and increase to generalizability of findings to this wider area. This resulted in the inclusion of five relevant studies conducted in Iran.”

**Thank you so much for your valuable suggestions.**
